# Loss-of-function of *MIR172b* and prime editing of *SNB* reveal a regulatory module underlying cleistogamy in rice

**DOI:** 10.1093/plphys/kiag435

**Published:** 2026-06-25

**Authors:** Su-Hyeon Shim, Rihua Piao, Dong-Yoon Seo, Sang-Kyu Lee, So Young Park, Backki Kim, Hyun-Sung Leem, Dong-Hoon Jeong, Hee-Jong Koh, Jong-Seong Jeon

**Affiliations:** Graduate School of Green-Bio Science and Crop Biotech Institute, Kyung Hee University, Yongin 17104, Republic of Korea; Department of Plant Science, Seoul National University, Seoul 08826, Republic of Korea; Rice Research Institute, Jilin Academy of Agricultural Sciences, Gongzhuling 136100, China; Graduate School of Green-Bio Science and Crop Biotech Institute, Kyung Hee University, Yongin 17104, Republic of Korea; Graduate School of Green-Bio Science and Crop Biotech Institute, Kyung Hee University, Yongin 17104, Republic of Korea; Division of Life Science, Gyeongsang National University, Jinju 52828, Republic of Korea; Department of Life Science and Multidisciplinary Genome Institute, Hallym University, Chuncheon 24252, Republic of Korea; Department of Plant Science, Seoul National University, Seoul 08826, Republic of Korea; Department of Plant Bioscience and Life and Industry Convergence Research Institute, Pusan National University, Miryang 50463, Republic of Korea; Graduate School of Green-Bio Science and Crop Biotech Institute, Kyung Hee University, Yongin 17104, Republic of Korea; Department of Life Science and Multidisciplinary Genome Institute, Hallym University, Chuncheon 24252, Republic of Korea; Department of Plant Science, Seoul National University, Seoul 08826, Republic of Korea; Graduate School of Green-Bio Science and Crop Biotech Institute, Kyung Hee University, Yongin 17104, Republic of Korea

## Abstract

Cleistogamy, or self-fertilization of a closed flower, can limit unintended gene flow and may contribute to varietal purity in rice (*Oryza sativa*), with potential value for transgene containment. We previously identified the natural cleistogamous mutant *lodiculeless spikelet* (*ld*). Here, using map-based cloning, we show that *ld* carries a 4.6-kb deletion encompassing the entire *MICRORNA806a* (*MIR806a*) precursor and the upstream region of the *MIR172b* locus. Clustered regularly interspaced short palindromic repeats (CRISPR)/CRISPR-associated nuclease 9-mediated editing of the *MIR806a* or *MIR172b* locus demonstrated that the loss of accumulation of miR172b, but not miR806a, is responsible for the cleistogamy phenotype of *ld* plants. Small RNA sequencing confirmed that miR172b is nearly absent in *ld* mutants. Among the five *AP2*-like genes harboring miR172b target sites, only *SUPERNUMERARY BRACT* (*SNB*) transcripts accumulated to significantly higher levels in young *ld* panicles than the wild type. Prime editing of the miR172-binding site in *SNB* generated a miR172-resistant *SNB* transcript isoform that reproduced the vestigial lodicule phenotype, indicating that the derepression of *SNB* transcript accumulation is sufficient to alter lodicule development. Histological analysis of rice harboring the *GUS* reporter gene driven by the *MIR172b* or *SNB* promoter revealed a strong overlap between the *MIR172b* and *SNB* expression domains in developing lodicules, supporting their regulatory relationship. Together, these results identify the miR172b-mediated repression of *SNB* transcript abundance as an important regulatory module for lodicule development and cleistogamy in rice. The agronomically neutral *ld* alleles represent valuable genetic resources for developing cleistogamous rice cultivars.

## Introduction

Cleistogamy is a mode of pollination in which flowers remain closed, resulting in self-fertilization. In grasses, flower opening is primarily controlled by lodicules. These specialized organs swell rapidly at anthesis and generate the force needed to pry open the spikelet. When lodicules fail to swell, lack proper cellular structure, or are transformed into other organ types (such as palea-like tissue), the flower remains closed, leading to cleistogamy (Yoshida et al. 2007; Nair et al. 2010). Cleistogamy can limit outcrossing between cultivated species and their wild relatives, may contribute to varietal purity in autogamous crops, and has potential applications in reducing transgene flow and pathogen exposure during anthesis.

Cleistogamy has evolved in approximately 300 plant species from 60 families (Lord 1981). This trait is particularly prevalent in grasses, where several crop species such as barley (*Hordeum vulgare*), wheat (*Triticum aestivum*), and rice (*Oryza sativa*) have been studied to elucidate the genetic basis of cleistogamy. In barley, the *cleistogamy 1* (*cly1*) mutant exhibits tightly closed spikelets during anthesis due to defective lodicule function (Kurauchi et al. 1993; Turuspekov et al. 2004). This phenotype is caused by a mutation in the *Cly1* gene, an ortholog of Arabidopsis (*Arabidopsis thaliana*) *AP2*, which regulates floral organ identity (Nair et al. 2010). In wheat, three orthologs of *Cly1*, namely *TaAP2-A*, *TaAP2-B*, and *TaAP2-D*, are highly expressed in lodicules, and their transcripts are targeted for cleavage by the microRNA (miR) miR172 (Ning et al. 2013). These findings establish the miR172–AP2 regulatory module as a conserved mechanism controlling lodicule development and cleistogamy in grasses.

miRNAs are a class of small non-coding RNAs that negatively regulate target gene expression at the post-transcriptional level (Rogers and Chen 2013). Functional mature miRNAs, which are 20 to 24 nucleotides (nt) long, are responsible for the cleavage or translational repression of their target transcripts. Plant miRNAs play important roles in growth, development, and environmental responses. In rice, 604 pre-*MIRNA*s generating 738 mature miRNAs have been reported. Among these, miR172 is a highly conserved miRNA that targets the transcripts of *AP2-like* transcription factor genes (Aukerman and Sakai 2003). miR172 and its *AP2-like* targets play crucial roles in developmental phase transitions and floral organ identity (Chen 2004; Zhu and Helliwell 2011; Spanudakis and Jackson 2014; Teotia and Tang 2015). In Arabidopsis, *AP2* is predominantly expressed in sepals and petals, whereas miR172 is enriched in anthers and carpels (Chen 2004; Wollmann et al. 2010). The barley *cly1* mutant harbors a synonymous substitution at the miR172 target site within the transcript of the *AP2-like* gene *Cly1*, suppressing miRNA-guided cleavage and leading to cleistogamy, underscoring the importance of precise miR172-mediated regulation for lodicule development (Nair et al. 2010).

In rice, several AP2-like proteins have been characterized as crucial regulators of flower organ development, flowering time, and grain-related traits. One of these, SUPERNUMERARY BRACT (SNB), mediates the transition from a spikelet meristem to a floral meristem; its dysfunction leads to the formation of extra bract-like organs (Lee et al. 2007). SNB has also been implicated in the regulation of flowering time, grain size, and seed shattering (Lee et al. 2014; Jiang et al. 2019; Ma et al. 2019). INDETERMINATE SPIKELET 1 (IDS1) acts synergistically with SNB to regulate inflorescence architecture and floral meristem development (Lee and An 2012). The *snb ids1* double mutant produces more bracts, including rudimentary glumes and palea/lemma, as well as elongated lodicules and a reduced number of stamens compared to the wild type (WT) (Lee and An 2012). RICE STARCH REGULATOR 1 (RSR1) negatively regulates the expression of type I starch biosynthesis genes and affects grain size, amylose content, and heading date (Fu and Xue 2010; Sun et al. 2022). SHATTERING ABORTION 1 (SHAT1) is essential for abscission zone development and seed shattering (Zhou et al. 2012). Another AP2-like protein, SALT AND ABA RESPONSE ERF 1 (SAE1), regulates ABA signaling by directly activating *ABA-INSENSITIVE 5* expression, enhancing seed germination and salinity stress tolerance (Li et al. 2022b). Although the genes encoding these AP2-like proteins harbor predicted miR172-binding sites, and recent work has shown that the miR172a–*SNB* module contributes to disease resistance in rice (Wang et al. 2025), studies of rice cleistogamy mutants have not directly implicated specific *MIR172* family members or their *AP2-like* targets in lodicule-mediated floral opening.

Several cleistogamy mutants have been reported in rice. In *cl7* plants, weakened lodicule swelling results in cleistogamy despite otherwise normal floral organ development (Ni et al. 2014). The *cl7* phenotype is caused by a mutation in *DENSE AND ERECT PANICLE 2*, which encodes a conserved plant-specific protein with no domains of known function; however, *cl7* exhibits undesirable pleiotropic effects (Li et al. 2010). The *superwoman 1-cleistogamy 1* (*spw1-cls1*) mutant carries a missense mutation in the class-B MADS-box gene *SPW1* that converts lodicules into lodicule-glume mosaic organs, but its cleistogamy phenotype is abolished under cold conditions (Yoshida et al. 2007). The temperature-stable allele *spw1-cls2* maintains cleistogamy even in cold environments but shows lower fertility than the WT (Lombardo et al. 2017). These mutants provide valuable insights but are of limited use for agriculture and breeding due to pleiotropic defects and environmental instability. Moreover, the contributions of individual *MIR172* family members to rice lodicule development, particularly through natural regulatory variation, remain largely unexplored.

One natural cleistogamy mutant in rice, *lodiculeless spikelet* (*ld*), is characterized by markedly reduced lodicules (Maeng et al. 2006). The *ld* mutation has no deleterious effects on major agronomic traits, including plant height, panicle structure, and yield (Won et al. 1998). Genetic mapping positioned the *LD* locus at the distal end of chromosome 1, suggesting that the causal factor is not the rice ortholog to barley *Cly1* based on the syntenic relationship between the barley and rice chromosomes (Maeng et al. 2006). In this study, we narrowed down the *LD* locus to a 23-kb region containing a 4,572-bp deletion that spans the entire *MIR806a* locus and the promoter region of *MIR172b*. Analysis of knockout mutants of *MIR806a* or *MIR172b* generated via clustered regularly interspaced short palindromic repeats (CRISPR)/CRISPR-associated nuclease 9 (Cas9)-mediated editing demonstrated that miR172b, but not miR806a, is essential for lodicule development. Among five *AP2-like* genes with predicted miR172-binding sites, only the transcripts of *SNB* markedly accumulated in the young panicles of *ld* plants. A miR172-resistant *SNB* variant created by prime editing of the miR172 target site in *SNB* recapitulated the vestigial lodicule phenotype observed in *ld* plants, and histological staining of rice harboring promoter:*GUS* revealed strong *MIR172b* and *SNB* promoter activity in developing lodicules. Together, these results demonstrate that loss-of-function of *MIR172b* induces cleistogamy by derepressing *SNB*, and they reveal a naturally occurring deletion in the *MIR172b* promoter as a genetic basis for cleistogamy. These findings provide a framework for precision breeding strategies aimed at modulating floral opening in rice.

## Results

### Isolation of the rice autogamy gene *LD*

**Figure 1:**
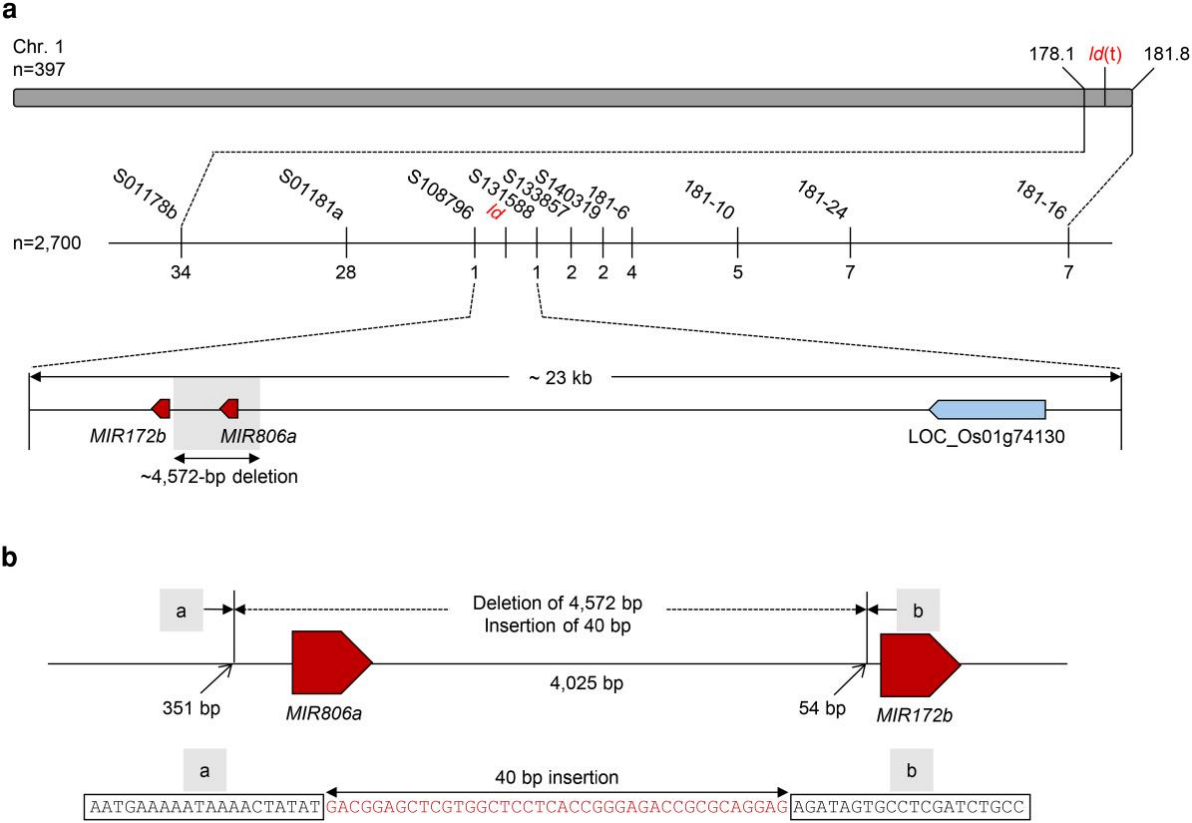
Map-based cloning of the *LD* locus. (a) Fine mapping and diagram of the *LD* locus. Primary mapping between the markers located at 178.1 Mb and 181.8 Mb on chromosome 1 (*n* = 397), and fine mapping (*n* = 2,700) between the markers S108796 and S131588. One annotated gene (LOC_Os01g74130) and two *MIRNA* loci (*MIR806a* and *MIR172b*) within the interval are highlighted. The rectangle indicates the 4,572-bp deleted region in *ld* identified by DNA walking. (b) Diagram of the deleted region in *ld-1* and the corresponding interval in the wild type. Gray boxes labeled “a” and “b” indicate the primers used to amplify across the junction of the deleted fragment. The 40-bp inserted sequence in *ld-1* is indicated.

We previously identified *ld*, a natural rice mutant with spikelets lacking normal lodicules, resulting in cleistogamy (Maeng et al. 2006). To investigate the causal mutation, we performed map-based cloning by constructing a high-resolution map using 1,027 F_2_ and 1,673 F_3_ segregating progeny from a cross between *ld* (in the *japonica* cultivar Ilpum background) and *indica* cultivar Milyang23. Fine mapping delimited the *ld* mutation to an approximately 23-kb genomic interval between STS markers S131588 and S108796 (Ji et al. 2012) (Fig. 1a). In silico analysis revealed that the region contains one predicted gene (LOC_Os01g74130) and two *MIRNA* loci (*MIR172b* and *MIR806a*) in the *japonica* reference genome (cultivar Nipponbare). We sequenced the 23-kb genomic region in the *ld* mutant and WT cultivar Ilpum and compared them to the sequence of the corresponding genomic region from the publicly available Nipponbare genome. This analysis identified a 4,572-bp deletion and a 40-bp insertion in *ld*; this deletion encompasses the entire *MIR806a* genomic region and the *MIR172b* promoter region (Fig. 1b). BLAST analysis of the 40-bp insertion sequence against the rice reference genome (IRGSP-1.0) revealed that 38 of 40 bp are identical to sequences within long terminal repeat (LTR) retrotransposon elements present at multiple genomic loci, indicating that the insertion represents filler DNA captured during double-strand break (DSB) repair via illegitimate recombination. The 23-kb region in WT Ilpum is identical to that of Nipponbare.

To further characterize the deleted region, we analyzed cis-regulatory elements in the *MIR172b* promoter (2.5 kb upstream of the pri-miRNA) using the PlantPAN 4.0 platform (https://plantpan.itps.ncku.edu.tw) (Chow et al. 2019; Chow et al. 2024). This analysis identified putative binding motifs for several transcription factor families, including AP2, MADS-box, B3, SBP (SPL), NF-YB, bZIP, NAC, and TCP transcription factors (Figure S1), suggesting that the deleted *MIR172b* promoter region contains multiple cis-regulatory elements important for proper *MIR172b* expression during floral organ development.

### CRISPR/Cas9-mediated functional validation of the *LD* gene

**Figure 2:**
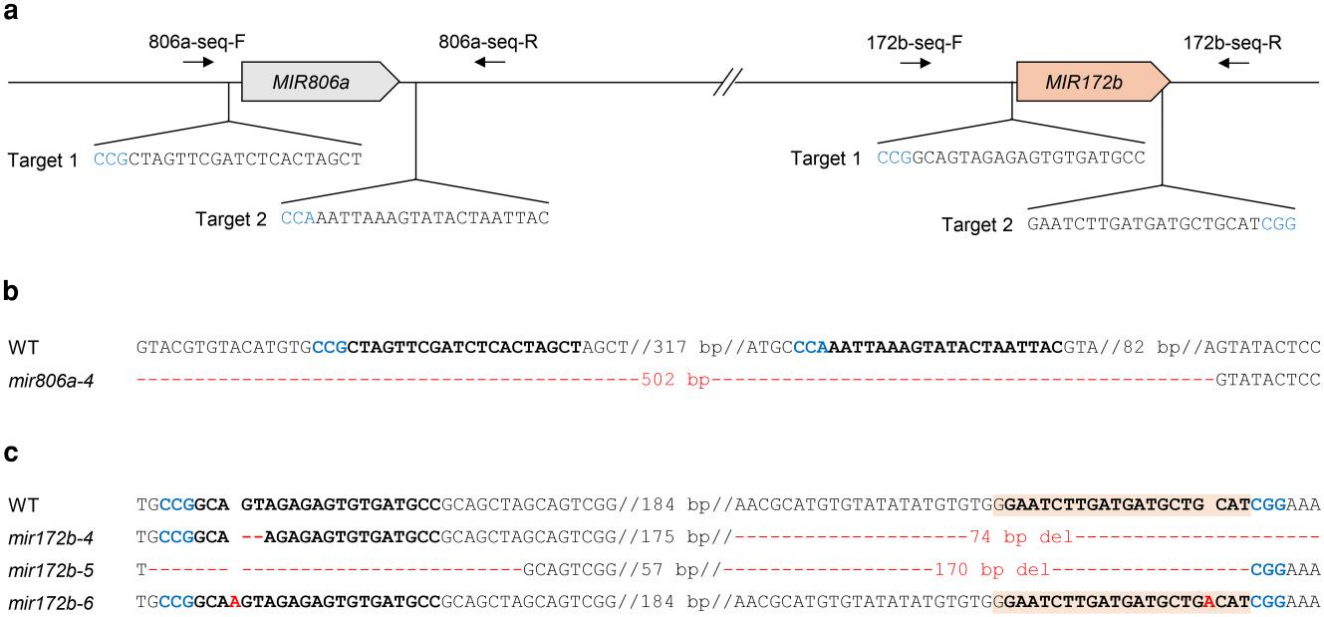
Generation of CRISPR/Cas9-mediated rice mutants defective in *MIR806a* or *MIR172b*. (a) Diagram showing the target sites for CRISPR/Cas9-mediated editing of the *MIR806a* or *MIR172b* locus. The target site upstream of each pri-*MIRNA* sequence is labeled “Target 1”, and the one downstream is labeled “Target 2”. PAMs are shown in blue. Primers used for genotyping are indicated with arrows. (b) Genomic sequence of the *mir806a-4* mutant harboring a deletion of the *MIR806a* locus. The deleted nucleotides in the mutant are shown as red hyphens. (c) Genomic sequences of the *mir172b* mutants carrying different deletions of the *MIR172b* locus. Portions of the *MIR172b* locus are deleted in *mir172b-4* and *mir172b-5*, and a single nucleotide is inserted within the mature miR172b region in *mir172b-6*. Deleted nucleotides are shown as red hyphens and the 1-bp insertion as bold red text; the mature miR172b region is highlighted in orange.

To determine which of the two miRNAs is responsible for the *ld* phenotype, we performed CRISPR/Cas9 multiplex genome editing to delete the sequences corresponding to the precursor of *MIR806a* or *MIR172b*. We designed two single guide RNAs (sgRNAs) for each *MIRNA* locus, one targeting the region immediately upstream of the precursor sequence and the other targeting either the downstream region or the mature miRNA site, to ensure complete functional disruption, potentially resulting in the deletion of the entire genomic region producing each precursor (Fig. 2a). We obtained 14 and 22 transgenic rice plants in the *japonica* cultivar Dongjin background expressing the CRISPR/Cas9 constructs targeting *MIR806a* or *MIR172b*, respectively. Among these, only one plant for *MIR806a* (designated *mir806a-4*) and two plants for *MIR172b* (designated *mir172b-4* and *mir172b-5*) carried large deletions in the respective locus based on Sanger sequencing of PCR products covering the intended targeted region (Fig. 2, b and c; Table S1). The *mir806a-4* mutant carries a 502-bp deletion that removes the entire *MIR806a* precursor sequence, including Target 1 and Target 2 (Fig. 2b). The *mir172b-4* mutant carries a 2-bp deletion at Target 1 and a 74-bp deletion covering Target 2, while *mir172b-5* carries a 31-bp deletion at Target 1 and a 170-bp deletion at Target 2 (Fig. 2c). In addition, we identified *mir172b-6*, with 1-bp insertions at both target sites (Fig. 2c). To avoid off-target effects, we analyzed all four sgRNA target sites using offTarget (Doench et al. 2016; http://skl.scau.edu.cn/offtarget) and listed all possible off-target sites (Off-score > 0.05) (Table S2). All *MIR806a* targets showed relatively low off-target potential, and no possible off-target sites were located within coding regions. By contrast, four putative off-target sites were identified for *MIR172b* targets, three of which possessed protospacer-adjacent motifs (PAMs) and relatively high Off-score values (> 0.1). We confirmed that no unintended editing occurred at any of the three putative off-target sites in *mir172b-4* or *mir172b-5* (Table S2; Figure S2).

**Figure 3:**
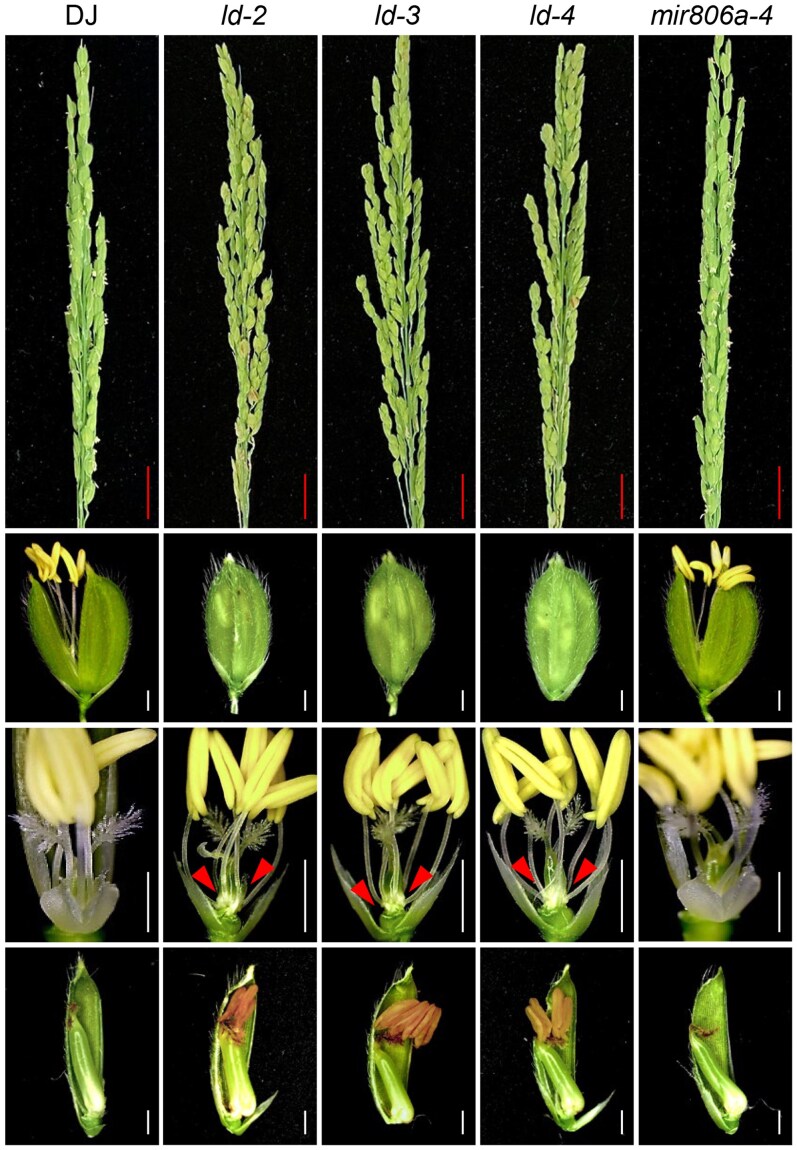
Phenotypic analysis of CRISPR/Cas9-mediated *mir806a* and *mir172b* mutants. Representative photographs of mature panicles, spikelets, and floral organs at anthesis and developing seeds (top to bottom) from WT Dongjin, *ld-2* (*mir172b-4*), *ld-3* (*mir172b-5*), *ld-4* (*mir172b-6*), and *mir806a-4* plants are shown. Arrowheads represent missing lodicules in the *ld* mutants. Red scale bars, 20 mm; white scale bars, 2 mm.

All *mir172b* mutants showed a clear cleistogamy phenotype similar to that of *ld*, whereas *mir806a-4* plants had a normal spikelet morphology indistinguishable from WT Dongjin (Fig. 3). We therefore designated *mir172b-4*, *mir172b-5*, and *mir172b-6* as *ld-2*, *ld-3*, and *ld-4*, respectively, while the original *ld* mutant was designated as *ld-1*. The spikelets of all *mir172b* mutant lines lacked normal lodicules, whereas Dongjin and *mir806a-4* developed intact lodicules (Fig. 3). Cleistogamous flowers release pollen without opening. To confirm the cleistogamous phenotype of the *ld* mutants, we observed dehulled spikelets at 3 to 6 d after fertilization. Indeed, all *mir172b* mutant lines retained anthers inside the spikelet, producing normal seed set after anthesis (Fig. 3). These genotypes and phenotypes were stably inherited across subsequent generations.

### miR172b is specifically downregulated in *ld* mutants

To investigate whether the loss of miR172b accumulation underlies the *ld* phenotype, we sequenced and analyzed small RNAs from the young panicles of WT Ilpum and two mutant alleles (*ld-1* and *ld-2*) at stage Sp5, i.e. during inner organ formation. Four *MIR172* loci are annotated within the rice *MIR172* family, generating three mature miRNA species: miR172ad, miR172b, and miR172c. Because these mature miRNAs share highly similar sequences and differ in only a few terminal nucleotides, it is difficult to distinguish their individual abundance levels by RNA gel blot analysis or RT-qPCR. We therefore performed small RNA sequencing to accurately quantify the abundance of individual miR172 family members.

**Figure 4:**
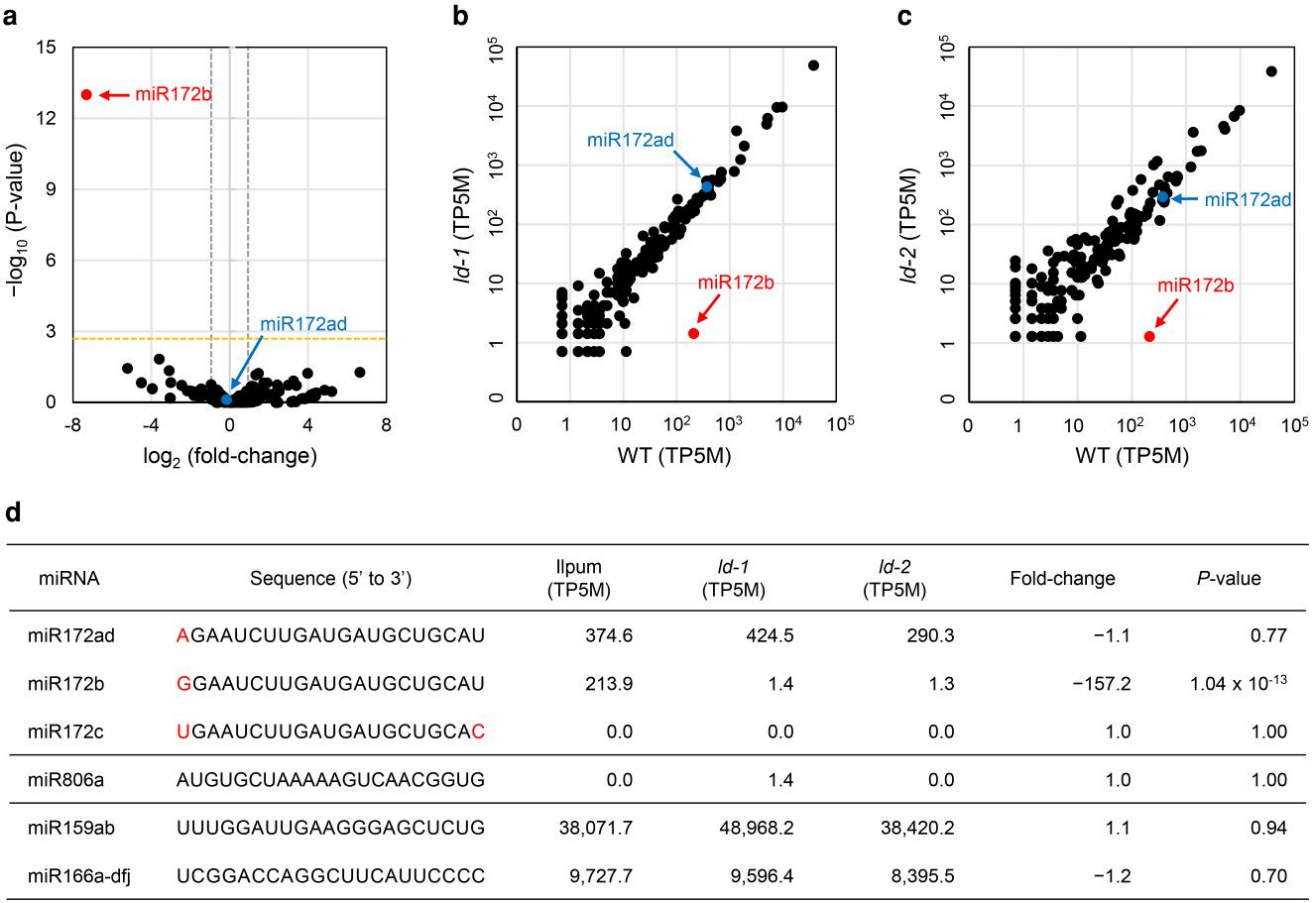
Characterization of miR172 family members accumulating in young panicles. (a) Volcano plot showing the fold-change relative to statistical significance (*P*-values) of normalized miRNA abundance between the *ld* mutants and WT Ilpum (IL). Gray dashed vertical lines indicate an absolute log_2_ (fold-change) threshold of 1, and the orange dashed horizontal line denotes *P*-value = 0.01. miR172ad and miR172b are shown as blue and red circles, respectively. (b), (c) Scatterplots of miRNA abundance in *ld-1* (b) or *ld-2* (c) vs. WT IL. miRNA levels were normalized as TP5M. miR172ad and miR172b are shown as blue and red circles, respectively. (d) Abundance, expressed as TP5M values, of selected miRNAs. miR172ad, miR172b, and miR172c represent mature miRNAs produced by different members of the rice *MIR172* family. miR159ab and miR166a-dfj served as controls. Red text indicates nucleotide differences among miR172 family members.

Mature miR172b was the only miR172 isoform showing a statistically significant difference in abundance between the WT and *ld* mutants (Fig. 4, a to c). The abundance of other known miRNAs, including miR172ad, was similar between the WT and *ld* mutants. In *ld-1* and *ld-2*, miR172b abundance was very low, with an average of only one or two reads detected, which may reflect trace contamination or sequencing noise (Fig. 4, b to d). Furthermore, miR172ad abundance was relatively high in the WT and *ld* mutants, whereas we detected no reads for miR172c in any sample regardless of genotype (Fig. 4d). Similarly, miR806a was undetectable in both the WT and *ld* mutants. Likewise, the abundance of control miRNAs such as miR159ab and miR166a-dfj was comparable between the WT and the two *ld* mutants (Fig. 4d). Collectively, these data confirm the notion that miR172b is the only mature miRNA that is specifically affected in *ld* mutants.

### *SNB* is a major target of miR172b during lodicule development

**Figure 5:**
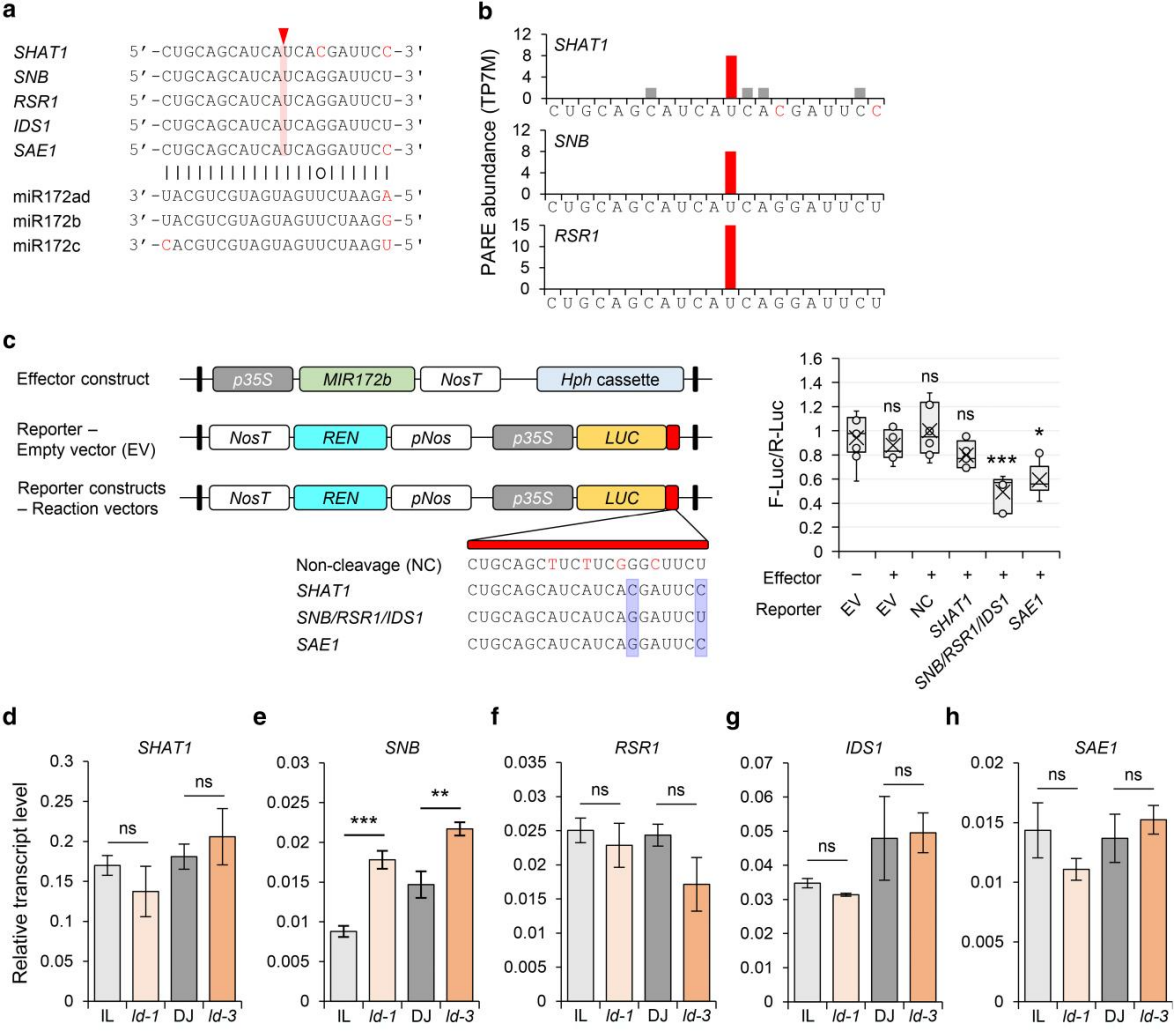
Validation of miR172b-mediated regulation of *AP2*-*like* genes. (a) Multiple nucleotide sequence alignment of the miR172 target sites complementary to the sequences of the indicated mature miR172 isoforms. Five putative miR172 target genes encoding AP2-like transcription factors and three mature miR172 isoforms are shown. Nucleotide differences among target sites or mature miR172 isoforms are shown in red. Vertical lines indicate perfect matches, and the G:U wobble pair is marked by a circle. The red arrow denotes the predicted miRNA-guided cleavage site. (b) Distribution of normalized PARE reads across the target sites. Red bars represent miR172-mediated cleavage events, while gray bars indicate randomly degraded RNA fragments. (c) Dual-luciferase assay to examine *MIR172b*-dependent regulation of *AP2-like* genes. Left, diagrams of the effector and reporter constructs. Each reporter construct consists of a miR172-binding sequence cloned into the 3′ UTR of the firefly luciferase (*LUC*) gene. Black bars, T-DNA borders; red boxes, 3′ UTRs of the *LUC* gene where the miR172 target sites were inserted. Right, relative luciferase activity in *N. benthamiana* leaves co-infiltrated with the effector and reporter constructs. Values are means ± SEM. Student's *t*-tests were performed (*n* = 6). *, *P* < 0.05; ***, *P* < 0.001. (d) to (h) RT-qPCR of *AP2*-*like* transcript levels in young panicles of WT cultivars Ilpum (IL) and Dongjin (DJ) and the corresponding *ld* mutants *ld-1* and *ld-3* at stage Sp5. (d) *SHAT1*; (e) *SNB*; (f) *RSR1*; (g) *IDS1*; (h) *SAE1*. Expression levels were normalized to those of *OsUBQ5*. Values are means ± SEM (*n* = 4; each is a pooled panicle from 3 to 5 plants). Statistical significance of differences was assessed using two-tailed Student's *t*-tests against the respective WT. **, *P* < 0.01; ***, *P* < 0.001; ns, not significant.

To identify the miR172b target gene responsible for the defective lodicule development in *ld* mutants, we examined the sequences of the five *AP2-like* transcription factor genes in rice that contain canonical miR172 complementary sequences: *SHAT1* (LOC_Os04g55560), *SNB* (LOC_Os07g13170), *IDS1* (LOC_Os03g60430), *RSR1* (LOC_Os05g03040), and *SAE1* (LOC_Os06g43220) (Sunkar et al. 2005; Zhu et al. 2009; Lee et al. 2014) (Fig. 5a). Analysis of publicly available Parallel Analysis of RNA Ends (PARE) datasets (Wu et al. 2009) detected clear miR172-guided cleavage signatures for *SHAT1*, *SNB*, and *RSR1* transcripts. Cleavage signatures for *IDS1* and *SAE1* could not be reliably detected in the available PARE libraries, likely due to the low transcript abundance of these genes in the tissues profiled, which would limit the sensitivity of cleavage product detection (Fig. 5b).

To more directly assess whether miR172b specifically regulates transcript abundance for these *AP2*-*like* genes, we performed Dual-luciferase reporter assays in *Nicotiana benthamiana* leaves. We cloned each representative miR172 target site downstream of the firefly luciferase (*LUC*) reporter gene, driven by the cauliflower mosaic virus *35S* promoter (*p35S*), with *Renilla* luciferase (*REN*) driven by the *Nopaline synthase* (*Nos*) promoter serving as an internal control. Because *SNB*, *RSR1*, and *IDS1* transcripts share an identical miR172-binding sequence, we generated three reporter constructs for *SHAT1*, *SNB/RSR1/IDS1*, and *SAE1*, respectively. A non-cleavable (NC) reporter containing deliberate mismatches in the miR172-binding site served as a negative control (Fig. 5, b to c). Co-infiltration of the effector construct *p35S:MIR172b* with either the empty vector (EV) or NC reporter resulted in relatively high relative LUC/REN values, comparable to that of infiltration with EV alone, confirming that the mutated miR172-binding sequence abolished miR172 recognition (Fig. 5c). By contrast, co-infiltration of *35S:MIR172b* with the reporters carrying the *SNB/RSR1/IDS1* or *SAE1* target site resulted in significantly lower LUC/REN values, indicating effective miR172b-mediated post-transcriptional repression of these transcripts (Fig. 5c). Infiltration of the *SHAT1* reporter construct did not result in a significant drop in LUC/REN values, indicating that repression was not detected in the heterologous system used in the Dual-luciferase assay under our conditions (Fig. 5c).

We next examined endogenous transcript levels of the five *AP2-like* genes in WT Ilpum and Dongjin and the corresponding mutants *ld-1* and *ld-3* to determine which targets become misregulated upon the loss of miR172b. Because lodicule development is initiated at an early stage of floral organ differentiation, we collected young panicles at the Sp5 stage from *ld* mutants and the corresponding WT plants (Itoh et al. 2005). RT-qPCR using gene-specific primers (Table S1) revealed that *SHAT1*, *IDS1*, *RSR1*, and *SAE1* transcript levels were unchanged in the *ld* mutants relative to the WT controls. By contrast, *SNB* transcript levels were markedly elevated in both *ld-1* and *ld-3* plants (Fig. 5, d to h). These results indicate that *SNB* is a major downstream target subjected to derepression in miR172b-deficient mutants. The enhanced accumulation of *SNB* transcripts is strongly associated with reduced lodicule size and the failure of floret opening, resulting in cleistogamy.

### miR172-mediated suppression of *SNB* expression is required for lodicule development

**Figure 6:**
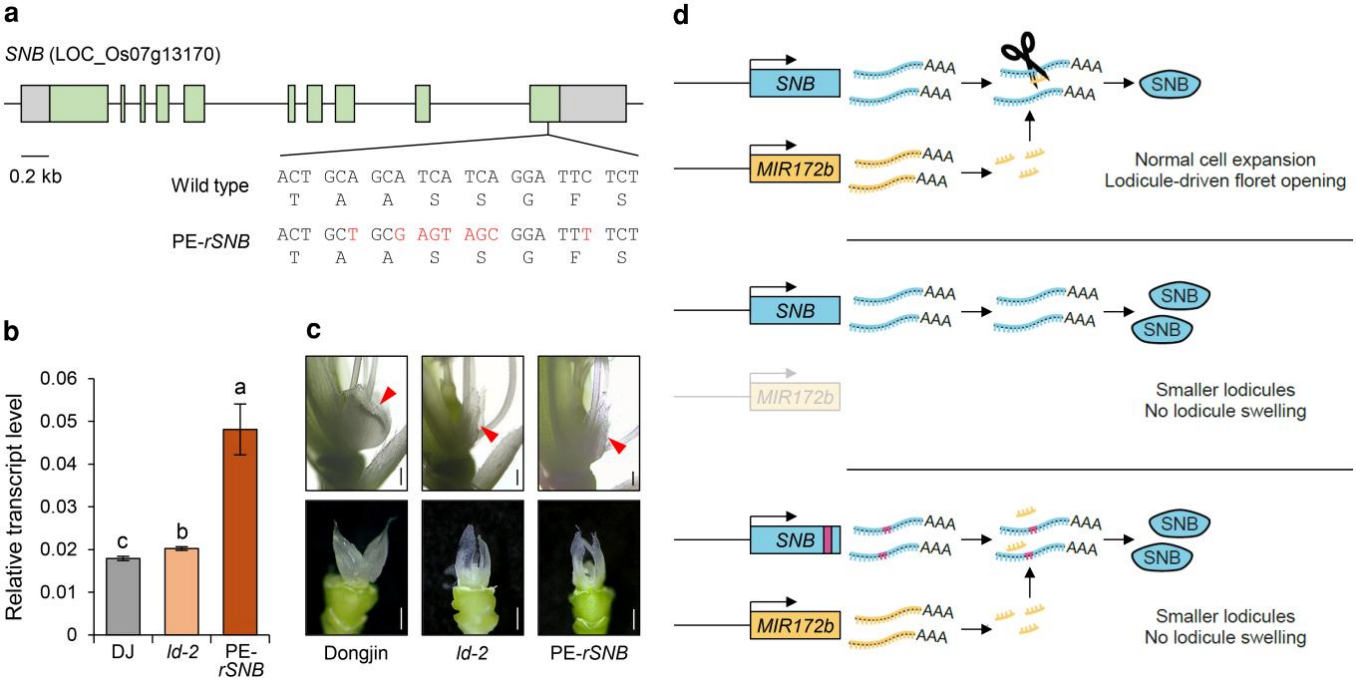
Characterization of an *rSNB* line generated by prime editing. (a) Diagram of the *SNB* locus and location of the edited sequence in the *rSNB* line. Green boxes, exons; gray boxes, UTRs. Red letters mark nucleotide substitutions introduced by prime editing. (b) Relative *SNB* transcript levels in young panicles of the indicated genotypes at stage Sp5. Values are means ± SEM. Different letters indicate statistically significant differences based on ANOVA (*P* < 0.05). (c) Magnified views of reproductive organs from WT Dongjin, *ld-2*, and PE-*rSNB* plants. Red arrowheads indicate lodicules. Black scale bars, 200 μm; white scale bars, 5 mm. (d) Proposed model for the miR172b–*SNB*-mediated regulation of lodicule development and cleistogamy in rice. In the wild type, miR172b represses *SNB* expression via mRNA cleavage in developing lodicules, enabling normal cell expansion and lodicule-driven floret opening at anthesis. In *ld* mutants lacking miR172b, or in PE-*rSNB* plants producing a miR172-resistant *SNB* transcript, *SNB* mRNA escapes miR172-mediated cleavage and accumulates ectopically, leading to smaller or vestigial lodicules. The failure of lodicule swelling prevents palea/lemma separation, resulting in cleistogamy. In addition, stronger or spatially expanded *SNB* derepression (as observed in PE-*rSNB*) disrupts panicle exsertion.

To validate the role of *SNB* in rice lodicule development, we generated a prime editing–mediated miR172-resistant form of *SNB*, designated PE-*rSNB*, in which the miR172-binding site was mutated to prevent complementarity while preserving the intact amino-acid sequence of SNB (Fig. 6a; Figure S3a). Among the five T_0_ plants obtained, we identified three *SNB/rSNB* heterozygotes without byproducts and subsequently obtained transgene-free homozygous *rSNB* mutant plants in the T_1_ generation (Figure S3b). The progeny of these homozygous plants were used for phenotypic analysis.

To confirm the accumulation of *rSNB* transcripts, we isolated total mRNA from young panicles at the Sp5 stage and performed RT-qPCR using *SNB*-specific primers (Table S1). *SNB* transcript levels were significantly higher in PE-r*SNB* than in WT Dongjin, suggesting that miR172-mediated post-transcriptional repression was disrupted in the mutant (Fig. 6b). Examination of mature flowers revealed that the lodicules from PE-*rSNB* plants were much smaller than the WT and vestigial in nature, similar to those of the *ld* mutants (Fig. 6c; Figure S4). Although lodicules were not visible in the *ld* mutants at low magnification (Fig. 3), closer inspection confirmed the presence of vestigial lodicules with an abnormal morphology in *ld* plants, similar to those in PE-*rSNB* (Fig. 6c; Figure S4). In addition to reduced lodicules, PE-*rSNB* plants exhibited a panicle enclosure phenotype in which panicles failed to emerge from the flag leaf sheath (Figure S5f). This phenotype resembles that of the *shortened uppermost internode 4* (*sui4*) mutant, which carries a missense substitution (Ser to Thr) in the miR172 target site of *SNB* (Ji et al. 2019). This panicle enclosure phenotype was not observed in any *ld* mutant, potentially due to the greater accumulation of *SNB* transcripts in PE-*rSNB* compared to *ld-2* (Figs. 3 and 6b). This additional phenotype in PE-*rSNB* plants suggests that stronger or spatially extended *SNB* derepression may occur and that the loss of regulation by other miR172 isoforms in tissues where they are normally active may also contribute to the enhanced defects.

To investigate the tissue-specific expression patterns of *MIR172b* and *SNB*, we generated *pMIR172b:GUS* and *pSNB:GUS* reporter lines containing 2.5-kb and 2-kb promoter fragments, respectively, driving the *GUS* reporter and performed GUS staining of homozygous T_3_ lines. We detected no GUS signal in the leaves or internodes of either line, although weak signals appeared in the nodes of *pSNB:GUS* plants (Figure S6). However, broad but weak GUS signals were detected throughout young panicles in both lines at stages Sp5 and Sp7, corresponding to the initiation and completion of lodicule primordium development, respectively (Figure S6). Strong GUS signals were observed specifically in the lodicules of mature flowers (Figure S6). These results indicate that *MIR172b* and *SNB* are co-expressed in expanding lodicules. These spatial expression patterns are consistent with a regulatory relationship between miR172b and *SNB* during lodicule development.

### Agronomic traits of *ld* and PE-*rSNB* mutants

Because cleistogamy limits floral opening and may reduce unintended outcrossing, cleistogamous rice has potential value for certain breeding applications. Previous studies have shown that the grain yield of the original *ld* (now *ld-1*) mutant was comparable to that of the WT (Won et al. 1998; Maeng et al. 2006). To evaluate the agronomic suitability of all *ld* mutants, we examined plant height, tiller number, mature grain number per panicle, grain morphology, and thousand-grain weight (TGW) (Figure S5). All plants were of similar height regardless of genotype, and tiller number and mature grain number per panicle were also unchanged among lines (Figure S5, a to c, e, and f). Grains were slightly shorter in all *ld* mutants compared to the respective WT plants; however, this decrease was offset by a modest increase in grain width, resulting in no significant change in TGW (Figure S5, d and g). The slight change in grain shape may reflect physical confinement of developing grains within closed spikelets.

## Discussion

In this study, we demonstrated that the cleistogamous phenotype of rice *ld* mutants results from loss-of-function of *MIR172b* caused by a deletion in its promoter and that derepression of *SNB* underlies their reduced lodicule growth. Fine mapping and sequencing analysis revealed a ∼4.6-kb deletion in the *ld-1* mutant that removes the entire *MIR806a* locus and the promoter region upstream of *MIR172b* (Fig. 1; Figure S1). Through the generation of CRISPR/Cas9-mediated loss-of-function mutants of *MIR806a* or *MIR172b*, we confirmed that the loss of miR172b, but not miR806a, underlies the cleistogamy phenotype of the mutants (Figs. 2 and 3). Small RNA sequencing further demonstrated that miR172b abundance was reduced to near-background levels (∼157-fold reduction) in *ld* mutants (Fig. 4), while all other miRNAs remained unchanged, providing strong evidence that the promoter deletion effectively abolishes *MIR172b* expression in vivo. Although the specific cis-regulatory elements removed by the ∼4.6-kb deletion remain to be defined, the strong reduction of miR172b abundance implies a direct causal link between promoter loss and miR172b deficiency. Functional dissection of individual regulatory elements within the deleted region will be an important direction for future investigation. In the same analysis, miR806a was undetectable even in WT plants (Fig. 4). This is consistent with the previous suggestion that *MIR806a* behaves more like a short-interacting RNA-generating locus rather than a canonical *MIRNA* gene; in fact, miR806a is no longer annotated as a *bona fide* miRNA in miRBase (Jeong et al. 2011; Kozomara et al. 2019).

Notably, the ∼4.6-kb deletion in *ld-1* is accompanied by a 40-bp insertion at the breakpoint junction. Of these 40 base pairs, 38-bp are identical to LTR retrotransposon sequences dispersed throughout the rice genome. The co-occurrence of a large deletion with a short LTR-derived filler DNA insertion is a structural hallmark of illegitimate recombination (Gorbunova and Levy 1997; Ma et al. 2004). During DSB repair, exposed 3′ single-stranded overhangs can transiently invade ectopic genomic templates sharing short microhomology via synthesis-dependent strand annealing, resulting in the incorporation of short filler sequences at the repair junction (Gorbunova and Levy 1997; Salomon and Puchta 1998). Given that LTR retrotransposon sequences constitute approximately 22% of the rice genome (Ma et al. 2004), these sequences represent highly accessible ectopic templates for filler DNA capture. This analysis indicates that the deletion in the *ld-1* mutant arose through illegitimate recombination rather than through precise transposon excision or homologous recombination between flanking repeats.

Previous work in barley revealed that cleistogamy can result from mutations in the miR172-binding sites of transcripts from the *AP2-like* gene *Cly1* (Nair et al. 2010), suggesting that disrupted miR172−*AP2*-mediated regulation impairs lodicule development. Because miR172 and *AP2-like* targets are highly conserved in grasses, we examined five *AP2*-*like* genes in rice that harbor canonical miR172-binding sequences. Analysis of PARE data identified in vivo miR172-guided cleavage between the adenosine at position 10 and uracil at position 11 (of miR172b) for the *SHAT1*, *SNB*, and *RSR1* transcripts (Fig. 5, a and b). Dual-luciferase assays showed that LUC activity derived from the *p35S:LUC* reporter with an added miR172-binding site from *SNB/RSR1/IDS1* or *SAE1* at the 3′ UTR of *LUC* declined upon *MIR172b* expression, whereas a reporter with the miR172-binding site from *SHAT1* did not respond to the presence of miR172b, despite its high sequence complementarity to miR172b (Fig. 5, a and c). Among the five *AP2*-*like* genes, *SHAT1* is also the closest rice ortholog to barley *Cly1*, further suggesting that it might be a strong miR172 target. Analysis of PARE data confirmed that *SHAT1* cleavage occurs in vivo, indicating that the heterologous assay system may not fully recapitulate endogenous regulation. Importantly, *SHAT1* transcript levels did not increase in the *ld* mutants, indicating that *SHAT1* is not the primary mediator of cleistogamy (Fig. 5d). The absence of clear cleavage signatures for *IDS1* and *SAE1* in the analyzed PARE datasets does not exclude the possibility of miR172-mediated regulation, as detection sensitivity depends on transcript abundance and tissue specificity. In addition, plant miRNAs can repress the expression of their target genes through translational inhibition without producing cleavage signatures. By contrast, only *SNB* transcripts significantly accumulated in the young panicles of *ld* mutants (Fig. 5e). Collectively, these findings support the notion that *SNB* is a major functional target of miR172b during lodicule development.

Previous studies analyzing SNB function through knockout or constitutive overexpression did not report a cleistogamy phenotype (Lee et al. 2007, 2014; Jiang et al. 2019; Ma et al. 2019), suggesting that simple loss- or gain-of-function approaches may not fully capture the regulatory context of *SNB* during lodicule development. Therefore, we designed a prime-edited miR172-resistant *SNB* allele (PE*-rSNB*) that disrupts the miR172-binding site while preserving the intact protein sequence to clarify the molecular function of SNB under post-transcriptional regulation by miR172 (Fig. 6a; Figure S3a). PE-*rSNB* reproduced the vestigial lodicule phenotype seen in *ld* mutants, but also exhibited panicle enclosure, characterized by a failure of panicle exsertion from the flag leaf sheath. This phenotype was absent from *ld* mutants and resembled that of the *sui4* mutant, which carries a missense mutation in the miR172-binding site of *SNB* (Fig. 6, a and c; Figure S5, e and f) (Ji et al. 2019). These contrasting phenotypes indicate that the magnitude, spatial pattern, and timing of *SNB* derepression differ between *ld* and PE-*rSNB* plants. *SNB* transcript levels were moderately higher (1.2–2-fold) in *ld* mutants but were significantly higher (∼3-fold) in PE-*rSNB* plants than in the WT (Figs. 5e and 6b), suggesting a dosage effect, whereby mild derepression specifically reduces lodicule growth, whereas stronger or ectopic derepression disrupts panicle exsertion. Additionally, *SNB* transcripts produced by PE-*rSNB* plants likely escape repression by other miR172 isoforms (miR172ad and miR172c), resulting in derepression in tissues where these isoforms normally function, an escape mechanism that is absent from *ld* mutants. Although *IDS1* and other *AP2-like* genes harbor canonical miR172-binding sites and have been implicated in floral development, our genetic data indicate that derepression of *SNB* alone is sufficient to induce vestigial lodicule formation and cleistogamy. The PE-*rSNB* lines reproduced the lodicule defects observed in *ld* mutants without modifications of other *AP2* loci, supporting *SNB* as a major functional mediator of the phenotype. Nevertheless, we cannot exclude potential contributions from additional miR172 targets, such as *IDS1*, under specific developmental contexts, which warrant further investigation.

SNB is known to regulate spikelet meristem determinacy, and our results suggest that it also contributes to panicle architecture or stem elongation (Lee et al. 2007, 2012). This broader developmental role is consistent with the panicle enclosure phenotypes observed in both PE-*rSNB* and *sui4* plants (Ji et al. 2019). While miR172b-mediated regulation of *SNB* expression is clearly important for lodicule development, its downstream molecular mechanisms remain largely unresolved. The reduced lodicule size observed in *ld* mutants indicates impaired lodicule expansion; however, the cellular basis underlying this phenotype, such as altered cell proliferation, cell expansion, or cell identity, remains unresolved. Because AP2-type transcription factors can regulate developmental programs in diverse contexts (Zhao et al. 2019), SNB may influence pathways affecting lodicule growth. Detailed histological and cytological analyses will be required to distinguish among these possibilities. Identifying direct SNB target genes through chromatin immunoprecipitation followed by sequencing or DNA affinity purification followed by sequencing, together with transcriptome profiling of *ld* mutants across developmental stages, will also be essential in order to elucidate the SNB-dependent developmental program of lodicules. In addition, although the *GUS* reporter assays supported spatial overlap between *MIR172b* and *SNB* expression domains (Figure S6), they did not resolve the precise temporal dynamics of their expression during lodicule development. Higher-resolution stage-specific or quantitative analyses will be required to clarify the timing and hierarchy of this regulatory interaction.

A conceptual model summarizing the regulatory relationship between miR172b, *SNB* expression levels, and the resulting floral phenotypes in WT, *ld*, and PE-*rSNB* plants is shown in Fig. 6d. This model illustrates how the degree and spatial pattern of *SNB* derepression determine whether plants undergo normal anthesis (autogamy) or closed-flower selfing (cleistogamy) and explains the phenotypic differences between naturally occurring *ld* alleles and engineered *rSNB* mutants. Importantly, our findings point to the potential applications of these *ld* mutants for breeding cleistogamous rice cultivars. The *ld* alleles represent naturally occurring *MIR172b* loss-of-function mutations that result in a complete cleistogamy phenotype while maintaining normal plant growth, panicle exsertion, and grain yield across multiple backgrounds (Figure S5). By contrast, engineered *SNB* gain-of-function alleles, such as with PE-*rSNB* plants, exhibit undesirable pleiotropic defects. Thus, *ld* alleles provide a promising genetic resource for introducing cleistogamy into commercial rice cultivars.

In conclusion, our study demonstrates how the deletion of a single *MIR172* family member, *MIR172b*, induces cleistogamy through specific post-transcriptional derepression of the *AP2*-*like* gene *SNB*. Because *ld* alleles maintain normal agronomic traits while providing complete cleistogamy, these naturally occurring variants represent valuable genetic resources for developing cleistogamous rice cultivars, with potential applications for transgene containment and genetic purity management. However, understanding the extent to which cleistogamy reduces outcrossing under field conditions will require direct evaluation in agronomic settings.

## Materials and methods

### Plant materials and growth conditions

Seeds of *japonica* rice (*O. sativa*) cultivars Ilpum and Dongjin, the *indica* cultivar Milyang23, and mutant lines were surface sterilized and cultured on half-strength Murashige and Skoog medium for 10 d under a 14-hour light/10-hour dark photoperiod at 25℃. The seedlings were transferred to soil. Rice plants were grown in either a growth chamber under a 14-hour light/10-hour dark photoperiod and a 30/20 ℃ (day/night) cycle or in an experimental field under natural conditions.

### Map-based cloning

The *ld-1* mutant used in this study was originally identified as CL-SNU, a natural mutant from *japonica* rice germplasm in the Ilpum background (Won et al. 1998; Maeng et al. 2006). The *LD* locus was previously mapped to the distal region on the long arm of chromosome 1 (Maeng et al. 2006). In the current study, the *ld* mutant was crossed with *indica* cultivar “Milyang23′, and 1,027 F_2_ plants were used for fine mapping. To further narrow down the *LD* region, 1,673 F_3_ plants derived from F_2_ lines carrying recombination events within the interval were analyzed.

Genomic DNA was isolated from young leaves using a simple miniprep method (Chen and Ronald 1999). Ten STS markers were developed for fine mapping of the *LD* locus based on the DNA sequence differences between Milyang23 and the *japonica* rice subspecies (Ji et al. 2012). The full-length sequence of the approximately 23-kb mapping region was divided into segments to design specific primers to amplify genomic DNA from the *ld* mutant and the WT cultivar Ilpum. To identify the region harboring the mutation, a DNA walking strategy was employed using a DNA Walking SpeedUp Premix Kit (Seegene, Korea). The primers qLD-F1 and qLD-R1 were used for amplification (Table S1). The resulting amplicons were cloned and subjected to Sanger sequencing. The deleted region was confirmed using primers qLD-F2 and qLD-R2 (Table S1).

### Generation of additional *ld* mutants by CRISPR/Cas9 editing

To identify effective PAM sites and to avoid off-target effects, 20-bp gene-specific target sequences within the presumptive *LD* locus were selected using the CRISPRdirect web tool (Naito et al. 2015; https://crispr.dbcls.jp). To induce large deletions in each *MIRNA* locus, two sets of sgRNAs were designed to target *MIR806a* (5′-AGCTAGTGAGATCGAACTAG-3′ and 5′-GTAATTAGTATACTTTAATT-3′) or *MIR172b* (5′-GGCATCACACTCTCTACTGC-3′ and 5′-GAATCTTGATGATGCTGCAT-3′). To synthesize the tRNA-sgRNA1-tRNA-sgRNA2 cassettes, PCR fragments were amplified using primer sets L5AD5-F/806a-T1-R, 806a-T1-F/806a-T2-R, and 806a-T2-F/L5AD5-R for *MIR806a* and L5AD5-F/172b-T1-R, 172b-T1-F/172b-T2-R, and 172b-T2-F/L5AD5-R for *MIR172b*, with the pGTR plasmid (#63143, Addgene, MA, USA) used as the template (Table S1). The resulting DNA fragments were assembled using the Golden Gate method, as described by Xie et al. (2015), and subcloned into pRGEB32, a binary vector for multiplex genome editing. The final constructs were transformed into *Agrobacterium tumefaciens* strain LBA4404 and introduced individually into *japonica* rice cultivar “Dongjin” via Agrobacterium*-*mediated transformation (Jeon et al. 2000). Mutations at the sgRNA target sites were identified by Sanger sequencing of PCR amplicons generated with the primer sets 806a-seq-F/806a-seq-R and 172b-seq-F/172b-seq-R (Table S1). Transgene-free homozygous T_1_ mutants were isolated from the progeny of self-fertilized T_0_ plants, and T_2_ or T_3_ plants were used for all experiments.

### Assessment of the cleistogamy phenotype

To visualize plant growth and flowering phenotypes, field-grown plants and their panicles were harvested from each genotype at the anthesis stage and photographed using a Canon EOS 100D camera (Canon, Japan). Individual spikelets, florets, and lodicules from dehulled flowers were observed under a stereomicroscope (SZX16, Olympus, Japan). For detailed morphological observations, lodicules were examined under a TM-20B stereomicroscope (Sunny Optical Technology, Korea).

### Small RNA library construction and sequence analysis

Total RNA was isolated from the young panicles of WT Ilpum and *ld* plants using TRIzol reagent (Invitrogen, MA, USA), and 20–30-nt small RNAs were purified from a 15% (w/v) denaturing PAGE gel (Accerbi et al. 2010). Small RNA libraries were constructed using a NEXTFlex Small RNA-seq Kit v3 (Bioo Scientific, TX, USA) and sequenced on the Illumina MiSeq platform. Small RNA sequencing data were processed using the CLC Genomics Workbench version 11.0.1 (Qiagen, Germany). Rice miRNAs registered in miRBase Release 22 were used to identify mature miRNAs with perfect matches and to quantify their abundance (Kozomara and Griffiths-Jones 2014). Their abundance was normalized as transcripts per 5 million reads (TP5M). Differentially abundant mature miRNAs between WT and *ld* plants were identified using the R package EdgeR with the thresholds fold-change > 2 and *P*-value < 0.01. Raw reads from small RNA sequencing have been deposited in the Gene Expression Omnibus at the National Center for Biotechnology Information (NCBI) under accession number GSE174230.

### Analysis of PARE library data

To validate miR172-mediated target cleavage, publicly available PARE data were retrieved from the NCBI Gene Expression Omnibus (accession number GSM455939) and analyzed. PARE reads that mapped to target genes were obtained from the website https://mpss.meyerslab.ucdavis.edu/oryza-sativa.

### Dual-luciferase assay

For the effector construct, a binary vector containing the *MIR172b* primary *MIRNA* under the control of the cauliflower mosaic virus 35S promoter (*p35S*) was generated. An attB1-*MIR172b*-attB2 fragment was amplified from Dongjin genomic DNA and cloned into the entry vector pDONR201 using Gateway BP Clonase II Enzyme mix (Invitrogen, MA, USA). The resulting entry plasmid was recombined with the Gateway-compatible binary vector pGWB502 using Gateway LR Clonase II Enzyme mix (Invitrogen, MA, USA).

For the reporter constructs, the pGreen:dual-luc vector was used (#55206, Addgene, MA, USA), containing the *REN* reporter gene driven by the *Nopaline synthase* promoter (*pNos*) and a *p35S:firefly luciferase* (*LUC*) expression cassette. The 21-bp miR172-binding sites for five *AP2*-*like* genes (*SNB*, *RSR1*, *IDS1*, 5′-CUGCAGCAUCAUCAGGAUUCU-3′; *SHAT1*, 5′-CUGCAGCAUCAUCACGAUUCC-3′; *SAE1*, 5′-CUGCAGCAUCAUCAGGAUUCC-3′) and a non-cleavage control sequence (5′-CUGCUGCGAGUGACGGCUUUU-3′) were individually inserted into the 3′ untranslated region (3′ UTR) of *LUC* at the *Avr*Ⅱ and *Age*Ⅰ restriction sites. Effector and reporter constructs were individually introduced into Agrobacterium strain GV3101.

Effector–reporter pairs were co-infiltrated into the leaves of *Nicotiana benthamiana* plants following the method of Ma et al. (2012). Dual-luciferase assays were performed using leaf samples collected three days after infiltration as described by Liu et al. (2014). A Dual-Glo Luciferase Assay System (Promega, WI, USA) was used for lysis and luminescence measurements, and LUC and REN activities were quantified using a VICTOR Nivo Multimode Plate Reader (PerkinElmer, MA, USA). Three biological replicates, each with three technical replicates, were analyzed per genotype.

### mRNA isolation and transcript analysis

To quantify the expression levels of the five *AP2*-*like* genes predicted to be miR172b targets, total RNA was extracted from young panicles at the Sp5 stage using a FastPure Universal Plant Total RNA Isolation Kit (Vazyme, China). First-strand cDNA was synthesized using ReverTra Ace qPCR RT Master Mix (Toyobo, Japan). Quantitative PCR was performed using gene-specific primers (Table S1) and Prime Q-Master Mix (Genet Bio, Korea) on a Rotor-Gene 6000 Real-Time PCR system (Qiagen, Germany). The rice *UBIQUITIN 5* gene (*OsUBQ5*; LOC_Os01g22490) was used as an internal control (Table S1). For RT-qPCR, four independent biological replicates were analyzed, with each biological replicate consisting of pooled young panicles from 3 to 5 plants of the same genotype. Each biological replicate was assayed in three technical replicates, and the mean value of the technical replicates was used for statistical analysis. Statistical significance was assessed using two-tailed Student's *t*-tests comparing mutants to their respective WT controls. *P*-values < 0.05 were considered statistically significant. Data are presented as means ± standard error of the mean (SEM).

### Generation of a miR172-resistant *SNB* line by prime editing

To modify the miRNA172-binding site in the endogenous *SNB* locus while preserving the native amino-acid sequence, a 21-bp target region (5′-CTTCGTGGTCATGGCAAATGC-3′) was selected using the CRISPRdirect program (Naito et al. 2015; https://crispr.dbcls.jp). A pegRNA was designed to include a primer binding site (5′-TTTGCCATGA-3′) and a reverse transcription template (5′-AAAATCCGCTACTCGCAGCAGTAGGGAGTAATGGCAAAGGGGAGCCCTGCA-3′), conjugated to the evopreQ1 pseudoknot RNA motif (Figure S3a). The pegRNA was cloned into the enhanced pPE2 binary vector following the method of Li et al. (2022a). The resulting plasmid was introduced into *japonica* cultivar Dongjin by Agrobacterium-mediated transformation (Jeon et al. 2000). T_0_ transgenic plants were screened for editing by PCR using the primers SNB-qRT-F and SNB-qRT-R, followed by Sanger sequencing of the amplicons (Table S1). Transgene-free homozygous plants were subsequently isolated from the progeny of self-fertilized T_0_ plants, and T_1_ lines derived from these plants were used for analysis.

### Analysis of GUS transgenic rice

To analyze tissue-specific expression patterns, a 2.5-kb promoter fragment of *MIR172b* and a 2-kb promoter fragment of *SNB* were individually amplified using the primer pairs pMIR172b-F/pMIR172b-R and pSNB-F/pSNB-R, respectively (Table S1). The PCR fragments were cloned upstream of the *GUSplus* reporter gene in the binary vector pCAMBIA1305, and transgenic plants were generated by Agrobacterium-mediated transformation (Jeon et al. 2000). The resulting *pMIR172b:GUS* and *pSNB:GUS* transgenic plants were advanced for three generations to obtain homozygous lines. Various tissues (leaves, stems, young panicles, and flowers) were collected, fixed, and stained with 1 mM X-Gluc (Thermo Fisher Scientific, MA, USA) for GUS histochemical analysis following Shim et al. (2022). Stained samples were examined under an SZX16 stereomicroscope (Olympus, Japan).

### Analysis of agronomic traits

To analyze agronomic traits, eight individual plants per genotype grown in the paddy field were used to measure plant height and tiller number at the mature stage before harvest. Grain number per panicle was counted from three individual plants using three representative panicles per plant. Thousand-grain weight was measured from randomly selected grains after drying. Statistical comparisons were performed using two-tailed Student's *t*-tests.

### Statistical analysis

All datasets were generated from at least three biological replicates, each with three technical replicates, and error bars represent SEM or SD. Student's *t*-test or one-way ANOVA was used for statistical analysis as appropriate.

### Accession numbers

Raw small RNA sequencing reads generated in this study have been deposited in the NCBI Sequence Read Archive under accession number GSE174230.

## Supplementary Material

kiag435_Supplementary_Data

## Data Availability

The data underlying this article will be shared upon reasonable request to the corresponding author.
