# Supplementary material for: Loss-of-function of MIR172b and prime editing of SNB reveal a regulatory module underlying cleistogamy in rice
Source: Plant Physiol. 2026 Jun 25;201(3):kiag435. doi: 10.1093/plphys/kiag435 (PMC13360268; doi:10.1093/plphys/kiag435)

**Supplementary Figure S1.** Analysis of *cis*-regulatory elements in the *MIR172b* promoter.

Promoter sequences were analyzed using the PlantPAN 4.0 platform, revealing putative binding motifs for transcription factors associated with floral development and the phase transition, including MADS-box, AP2, B3, NAC, SBP (SPL), NF-YB, bZIP, and TCP transcription factors. In the scale at the top, the gray area indicates the deleted promoter region in *ld-1*, while the white area represents the portion that remains intact.


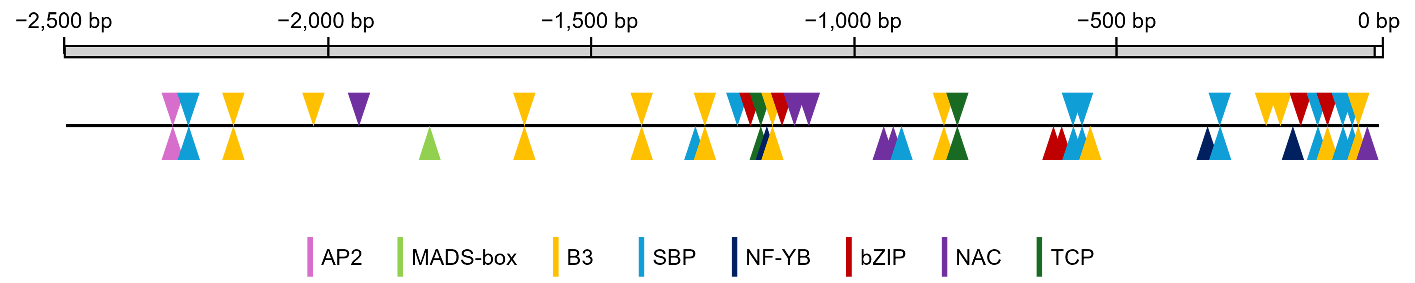


**Supplementary Figure S2.** Sanger sequencing of possible off-target sites in CRISPR/Cas9-mediated *ld* mutants.

**A B**

**
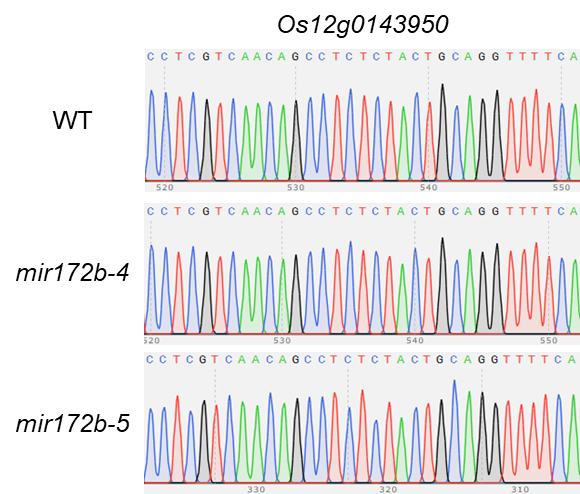

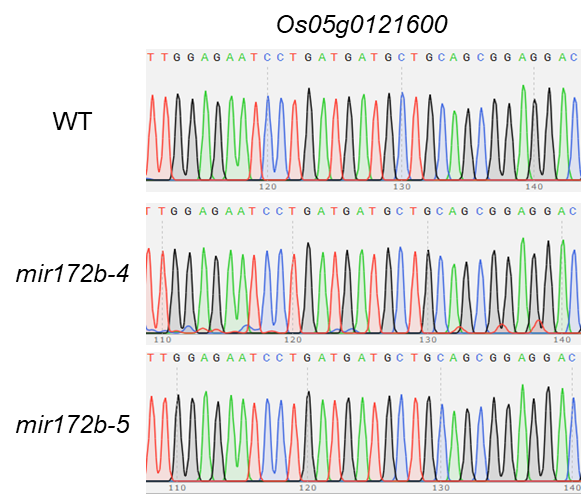
**

**C**

**
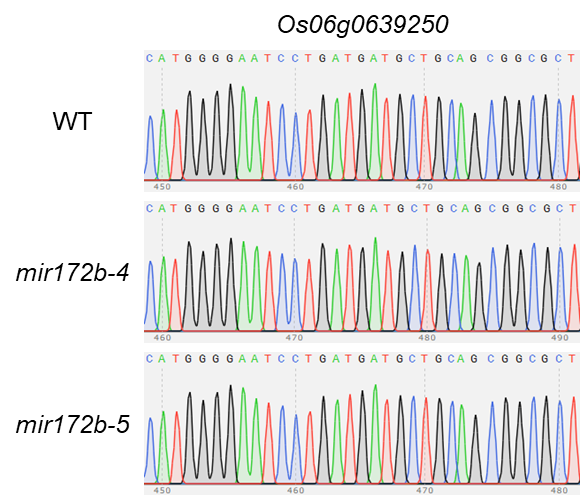
**

**Supplementary Figure S3.** The pegRNA sequence and Sanger sequencing of the PE-*rSNB* mutant.

**(A)** The pegRNA sequence for the PE-*rSNB* construct. The components are color-coded as follows: Blue represents the guide RNA scaffold, green indicates the HDV ribozyme, light green denotes the primer binding site, magenta indicates the reverse transcriptase (RT) template, orange denotes the 8-nucleotide linker, purple indicates the EvopreQ1 sequence for the pseudoknot, and yellow highlights the 20-nucleotide target sequence for nSpCas9. Intended mutations are indicated in gray. **(B)** Sanger sequencing results of the transgene-free homozygous T1 PE-*rSNB* mutant line. The orange box highlights the nSpCas9 target site, including the protospacer adjacent motif (PAM), while the blue box marks the site of miR172 binding with the intended homozygous mutation sites (•).

**A**

CTTCGTGGTCATGGCAAATGCGTTTTAGAGCTAGAAATAGCAAGTTAAAATAAGGCTAGTCCGTTATCAACTTGAAAAAGTGGCACCGAGTCGGTGCAAAATCCGCTACTCGCAGCAGTAGGGAGTAATGGCAAAGGGGAGCCCTGCATTTGCCATGAAGTAATACTTGACGCGGTTCTATCTAGTTACGCGTTAAACCAACTAGAAAGGCCGGCATGGTCCCAGCCTCCTCGCTGGCGCCGGCTGGGCAACATGCTTCGGCATGGCGAATGGGACTTTTTTTT

**B**


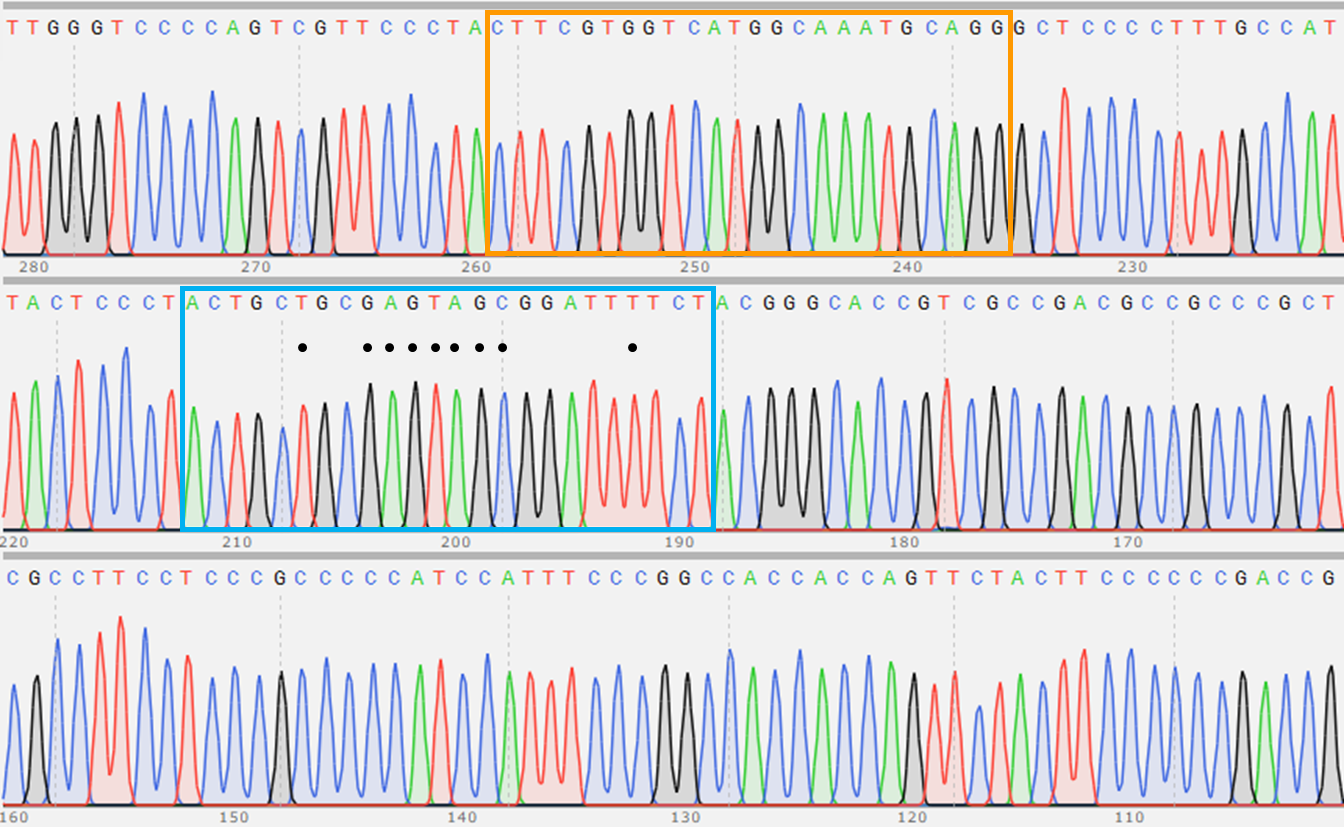


**Supplementary Figure S4.** Magnified views of lodicules in Ilpum and *ld-1* flowers.

Lodicules from wild-type (WT) Ilpum and *ld-1* spikelets at anthesis are shown. Red arrowheads indicate lodicules. Black scale bars, 200 μm; white scale bars, 5 mm.


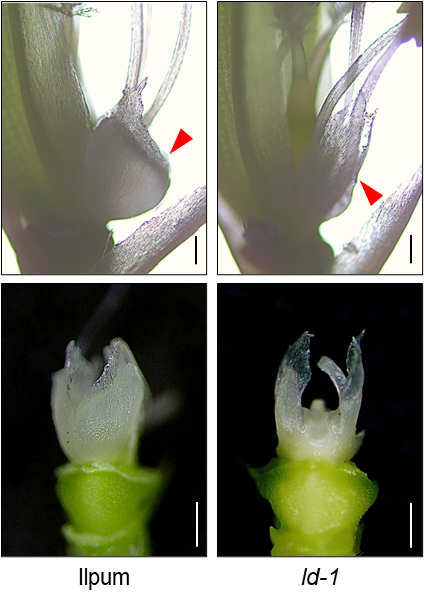


**Supplementary Figure S5.** Growth and agronomic traits of *ld* mutants and their respective WT controls.

**(A)** Plant height (in cm). **(B)** Tiller number per plant. **(C)** Number of mature grains per panicle. **(D)** Thousand-grain weight (in g). IL, Ilpum WT; DJ, Dongjin WT. Values are means ± standard deviation. ns, not significant (*P* > 0.05). **(E, F)** Representative photographs of *ld* mutants and their respective WT at the mature stage. Scale bars, 10 cm. **(G)** Representative photographs showing grain width and grain length for *ld* mutants and their respective WT controls. Scale bars, 3 mm.


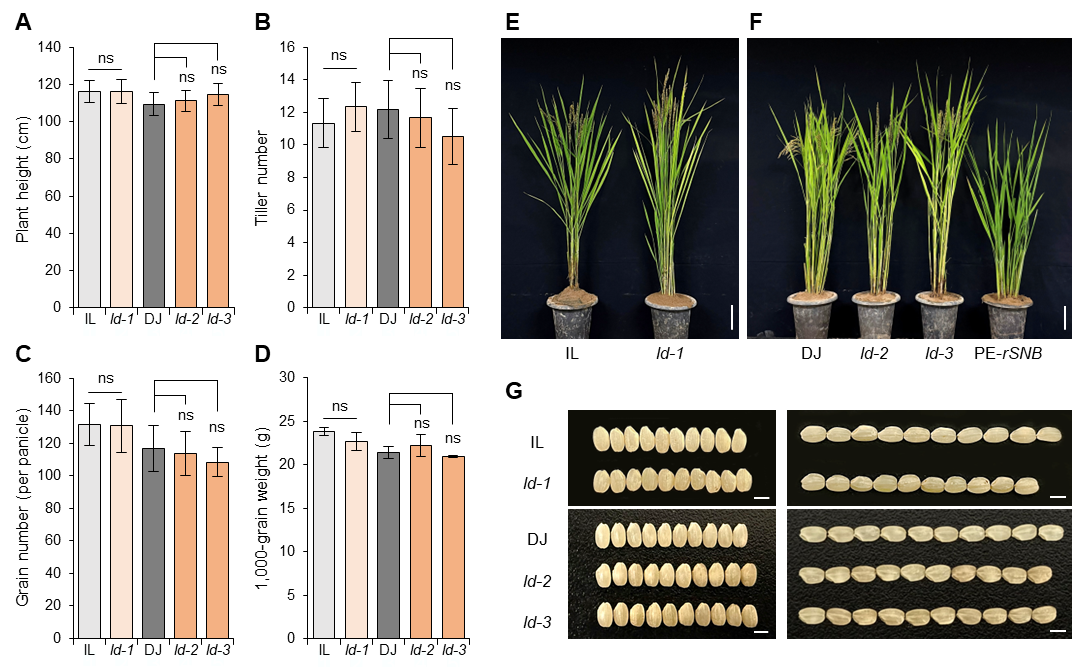


**Supplementary Figure S6.** GUS staining of *pMIR172b:GUS* and *pSNB:GUS* transgenic lines. From left to right: GUS staining patterns in leaves, stems, nodes, and young panicles (YP) at stages Sp5 and Sp7 and in mature flowers. Scale bars, 2 mm.


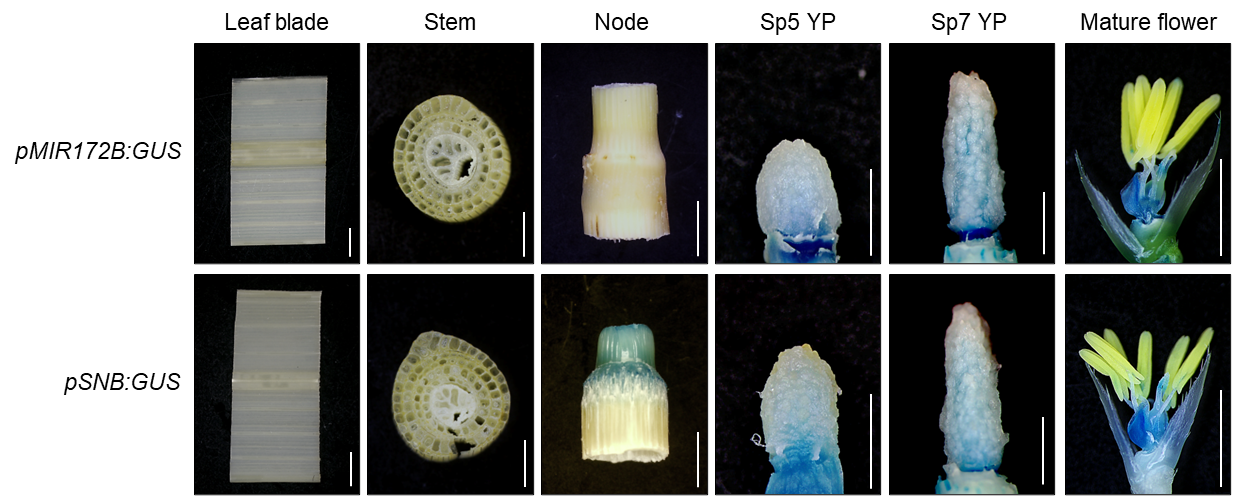

Supplement: kiag435_Supplementary_Data [file kiag435_supplementary_data.zip › Supplementary-Figures_submitted.docx]
