# Supplementary material for: Loss-of-function of MIR172b and prime editing of SNB reveal a regulatory module underlying cleistogamy in rice
Source: Plant Physiol. 2026 Jun 25;201(3):kiag435. doi: 10.1093/plphys/kiag435 (PMC13360268; doi:10.1093/plphys/kiag435)
Supplement: kiag435_Supplementary_Data [file kiag435_supplementary_data.zip › Supplementary-Tables_submitted.docx]

**Supplementary Table S1.** Primers used in this study.

| **Name** | **Sequence (5′ → 3′)** |
| --- | --- |
| qLD-F1 | GATGTAACCATCACCTTTACCG |
| qLD-R1 | CTATAAGTGCCACCTTGCTGCT |
| qLD-F2 | AGAGGGAGGGAGGATCACTC |
| qLD-R2 | CTTCGCTTGAGCTCCTCTGTT |
| L5AD5-F | CGGGTCTCAGGCAGGATGGGCAGTCTGGGCAACAAAGCACCAGTGG |
| L5AD5-R | TAGGTCTCCAAACGGATGAGCGACAGCAAACAAAAAAAAAAGCACCGACTCG |
| 806a-T1-F | TAGGTCTCCGTATACTTTAATTGTTTTAGAGCTAGAA |
| 806a-T1-R | ATGGTCTCAATACTAATTACTGCACCAGCCGGGAA |
| 806a-T2-F | TAGGTCTCCAGATCGAACTAGGTTTTAGAGCTAGAA |
| 806a-T2-R | ATGGTCTCAATCTCACTAGCTTGCACCAGCCGGGAA |
| 172b-T1-F | TAGGTCTCCATGATGCTGCATGTTTTAGAGCTAGAA |
| 172b-T1-R | ATGGTCTCATCATCAAGATTCTGCACCAGCCGGGAA |
| 172b-T2-F | TAGGTCTCCACTCTCTACTGCGTTTTAGAGCTAGAA |
| 172b-T2-R | ATGGTCTCAGAGTGTGATGCCTGCACCAGCCGGGAA |
| 806a-seq-F | CCCCATAGTACACTATTGAAGTAC |
| 806a-seq-R | AACATAACAGTACACAGTGTGTCC |
| 172b-seq-F | TGCCTTGCGTATTAAAGCTCCTTT |
| 172b-seq-R | CCTACACTAAATTATATGGCCAATC |
| SHAT1-qRT-F | CAACCGCTACAGCAGCTGCA |
| SHAT1-qRT-R | GACGATGAATGCAGCGATCTTG |
| SNB-qRT-F | AAATGCAGGGCTCCCCTTT |
| SNB-qRT-R | GGTGTCGCCTCCACCAGAATAG |
| RSR1-qRT-F | GGGGTAGCTGTTCCAAGCTG |
| RSR1-qRT-R | GTGGCGGCTTTGGAGAATCC |
| IDS1-qRT-F | AACACAGGCTTGGCAAATGC |
| IDS1-qRT-R | GGAAGTAGAAGGGGTGATCCG |
| SAE1-qRT-F | GCGCAGCAGAGCTACACAT |
| SAE1-qRT-R | ATGGGGAATCCTGATGATGCT |
| UBQ5-qRT-F | GACTACAACATCCAGAAGGAGTC |
| UBQ5-qRT-R | TCATCTAATAACCAGTTCGATTTC |
| pMIR172b-F | GGAACGTAGCGAGCTACGTAACAA |
| pMIR172b-R | CGACTGCTAGCTGCGGCATC |
| pSNB-F | GGTACCATGAACTGTCAAATGTTGAAC |
| pSNB-R | CCTAGGACTAACCAACCGCTCTCC |

**Supplementary Table S2.** Off-target analysis of *MIR806a* and *MIR172b* target sites in the CRISPR/Cas mutants. Protospacer-adjacent motifs (PAMs) are written in blue, and mismatched nucleotides are written in red. Possible off-target sites with an intact PAM in the coding region are highlighted in yellow.

| *MIR806a* Target 1 | | | AGCTAGTGAGATCGAACTAGCGG |  |  |  |
| --- | --- | --- | --- | --- | --- | --- |
| Chr. | Position | | Sequence | Off- score | Gene | Region |
| chr06 | 27606883 | | AGCTATTGGGATTGAACTAGCGG | 0.311 |  | Intergenic |
| chr11 | 16094743 | | AGATAGTGAGATCGAGCTAGAGG | 0.167 |  | Intergenic |
| chr03 | 27595075 | | AGATAATGAGATCGAGCAAGTGG | 0.111 |  | Intergenic |
| chr01 | 23540286 | | AGCTAGAGAGATTGGACTCGAGG | 0.082 |  | Intergenic |
| chr11 | 19592996 | | AGATGGTGAGATGGGACTAGCGG | 0.055 | Os11g0537375 | 5′−UTR |
|  |  | |  |  |  |  |
| *MIR806a* Target 2 | | | GTAATTAGTATACTTTAATTAGG |  |  |  |
| Chr. | | Position | Sequence | Off- score | Gene | Region |
| chr09 | | 13967421 | GTAATTAAGTTACTTTAATTTGG | 0.546 |  | Intergenic |
| chr02 | | 27348063 | GTAATTAGTATACAATAATTAGG | 0.358 |  | Intergenic |
| chr02 | | 9522250 | CTAGTTAGTATAATTTAATTTGG | 0.172 |  | Intergenic |
| chr12 | | 15996538 | GTATTTTGTATAATTTAATTTGG | 0.107 |  | Intergenic |
| chr09 | | 7660260 | CTATTTTGTATAATTTAATTTGG | 0.076 |  | Intergenic |
|  | |  |  |  |  |  |
| *MIR172b* Target 1 | | | GGCATCACACTCTCTACTGCCGG |  |  |  |
| Chr. | | Position | Sequence | Off- score | Gene | Region |
| chr12 | | 2163643 | GTCAACAGCCTCTCTACTGCAGG | 0.117 | Os12g0143950 | CDS* |
| chr06 | | 4524459 | GGCATCACTATCTTTAATCCCGG | 0.08 | Os06g0189200 | Intron |
| chr11 | | 18917833 | GGCATCACTATCTTTAATCCCGG | 0.08 |  | Intergenic |
| chr01 | | 30866379 | GGCCTTGCCCTCTCAACTGCCGG | 0.077 |  | Intergenic |
| chr05 | | 16769896 | GGCATCACTATTTCTACTGCCAG | 0.073 | Os05g0354300 | CDS* |
| chr11 | | 1224969 | TGCATCTCAATCTCTACTACCAG | 0.07 |  | Intergenic |
| chr04 | | 14694418 | GGCATGTCACTTTCTACTGAAGG | 0.059 |  | Intergenic |
| chr12 | | 3200433 | GCCATCGTGCTCTCTGCTGCCGG | 0.055 |  | Intergenic |
| chr12 | | 18037438 | CACATCAAACTCTCCAATGCCGG | 0.05 |  | Intergenic |
|  | |  |  |  |  |  |
| *MIR172b* Target 2 | | | GAATCTTGATGATGCTGCATCGG |  |  |  |
| Chr. | | Position | Sequence | Off- score | Gene | Region |
| chr02 | | 35143553 | GAATCTTGATGATGCTGCATCAG | 0.259 |  | Intergenic |
| chr05 | | 1156408 | GAATCCTGATGATGCTGCAGCGG | 0.16 | Os05g0121600 | CDS* |
| chr06 | | 25977504 | GAATCCTGATGATGCTGCAGCGG | 0.16 | Os06g0639250 | CDS* |
| chr09 | | 22881913 | GAATCTTGATGATGCTGCATCCG | 0.107 |  | Intergenic |
| chr05 | | 24519002 | AATTATTGATGATTCTGCATCGG | 0.097 | Os05g0498200 | 3′−UTR |
| chr08 | | 23544349 | GATGCTCGATGATGATGCATCGG | 0.067 |  | Intergenic |
| chr09 | | 19398095 | CGATCTTGTTGATGCTGCAGCGG | 0.061 |  | Intergenic |
| chr01 | | 42923944 | GAATCTTGATGATGCCACGTCAG | 0.06 |  | Intergenic |
| chr09 | | 19722092 | GATCCTTGATGATGATGAATCGG | 0.055 |  | Intergenic |
| chr09 | | 19725249 | GATCCTTGATGATGATGAATCGG | 0.055 |  | Intergenic |

* Coding sequence
